# Laparoscopic cholecystectomy in a resource limited setting: Factors associated with difficult surgeries at the National Referral Hospital, Bhutan

**DOI:** 10.1002/puh2.122

**Published:** 2023-09-17

**Authors:** Sangay Wangmo, Sonam Dargay, Sonam ChhodenR

**Affiliations:** ^1^ Faculty of Postgraduate Medicine Khesar Gyalpo University of Medical Sciences of Bhutan Thimphu Bhutan; ^2^ Department of Surgery Jigme Dorji Wangchuck National Referral Hospital Thimphu Bhutan

**Keywords:** evidence‐based medicine, patient safety, prediction score, surgical outcome

## Abstract

**Background:**

Prediction of difficult laparoscopic cholecystectomy may help in making necessary arrangements for optimal intraoperative requirements and postoperative care. This study was conducted to examine the factors associated with and outcomes of difficult laparoscopic cholecystectomy performed at the Bhutan's largest hospital in 2020.

**Methods:**

This was a cross‐sectional study with a convenience sampling method. Data on clinical features, ultrasonography and intraoperative factors of patients who underwent laparoscopic cholecystectomy were extracted from their medical records, investigation reports and intraoperative surgery note. Difficult laparoscopic cholecystectomy was defined on the basis of the duration of the surgery, injury to bile duct or artery, or conversion to open cholecystectomy. Data were double entered and validated in EpiData 3.1 and analysed in STATA 13.0.

**Results:**

Data from 134 patients were extracted. The mean age of the sample was 43 (±SD 13) years. “Difficult laparoscopic cholecystectomy” was reported in 83 patients (62%) and easy laparoscopic cholecystectomy in 51 patients (38%). Those patients having simple adhesions up to the body of the gall bladder were 1.6 times more likely to encounter difficult laparoscopic cholecystectomy (adjusted PR = 1.60, 95% CI 1.04–2.48, *p* = 0.034). The majority did not have any post‐operative complications (130 cases, 97%). The indications of laparoscopic cholecystectomy were symptomatic gall stone disease (129, 96%), acalculous cholecystitis (2, 1%) and gall bladder polyp (3, 2%).

**Conclusions:**

The proportion of difficult laparoscopic cholecystectomy is high, but the rates of post‐operative complications were minimal with no mortality or injury to bile duct or arteries.

## Graphical abstract

Laparoscopic cholecystectomy is one of the most common surgical procedures performed globally. Dissection of Calot's triangle during a routine elective laparoscopic cholecystectomy in Bhutan, 2022.

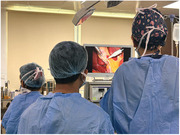

## BACKGROUND

Laparoscopic cholecystectomy is one of the most common surgical procedures performed globally and also one with frequent complications. In a systematic review of 233 studies between 2013 and 2016 that reported on complications related to laparoscopic cholecystectomy, 58% of the studies reported conversion to open cholecystectomy, 38% reported bile leak and 32% reported bile duct injury. Mortality related to laparoscopic cholecystectomy was reported in 38% of the studies included in the review [1]. Identifications of factors that determine or predict a difficult surgery are essential in making appropriate planning and preparation for surgery and post‐operative recovery period.

As access to surgical services improves in developing countries, laparoscopic cholecystectomy will be provided to an increasing number of people. There are patient factors, disease factors and operator skills that determine the difficulty in performing laparoscopic cholecystectomy. Although some laparoscopic cholecystectomy surgeries can be comfortably performed by trainee surgeons with appropriate supervision, some cases may be difficult even for the most experienced laparoscopic surgeon [2, 3].

“Difficult laparoscopic cholecystectomies” have been reported in terms of the duration of the surgery, spillage of bile, injury to bile ducts and arteries and the need to convert a laparoscopic into open cholecystectomy [4]. Some scoring systems are based on the dissection of the gall bladder, cystic pedicle and its associated adhesions [2, 5] or on the level of difficulty during the dissection of the gall bladder [6]. It is also reported on the basis of the duration of surgery, bile leakage injury to bile duct, injury to bile duct or artery and conversion to open cholecystectomy [7].

In resource‐limited settings, the identification of those patients who may encounter a difficult laparoscopic cholecystectomy will help in proper planning for surgery and arrangement of appropriate post‐operative care to improve surgical outcomes. In this article, we describe a real‐world scenario of the clinical, ultrasonography and intra‐operative factors associated with difficult laparoscopic cholecystectomy in Bhutan's largest hospital.

## METHODS

### Study design and study site

This was a cross‐sectional study conducted at the Department of Surgery, Jigme Dorji Wangchuck (JDW) National Referral Hospital, Thimphu, Bhutan. It is the apex referral hospital for surgical treatment of symptomatic gallstone diseases in Bhutan. This hospital has been offering laparoscopic surgeries since 1999 and cholecystectomy constitutes a significant share of clinical burden [8]. In 2020, there were six surgeons and seven surgery trainees in the department who performed laparoscopic cholecystectomy.

### Study population

Those ≥18‐year patients who underwent elective laparoscopic cholecystectomy at the JDW National Referral Hospital between 01 January and 31 December 2020 were included. The cases where laparoscopic surgeries were converted into open surgery due to equipment failure or emergency nature of surgery were excluded. There were no open cholecystectomy cases during the study period. In this study, data were collected only from those surgeries performed by surgeons and not by trainee surgeons.

### Sample size and sampling method

In 2019, based on the hospital records, approximately 200 laparoscopic cholecystectomies performed at the JDW National Referral Hospital. The pooled proportion of difficult laparoscopic cholecystectomies reported from centres in the South Asia region was 28.9% [4, 7, 9, 10].

The sample size was calculated for proportions with a confidence interval of 95%, margin of error of 0.05, variance or standard deviation 28.9%. After adjusting for sampling fraction ≥15% and finite correction for 200 surgeries performed in the previous year, allowing for a 5% dropout rate, the adjusted sample size was 141. A convenience sampling method was used to include data from all patients who underwent laparoscopic cholecystectomy at our centre.

### Data collection

Data were collected using a pro forma that was designed for the purpose of this study. This pro forma was pre‐tested among 10 patients who underwent laparoscopic cholecystectomy in December 2019. Changes were made to improve clarity on the specific variables that to be extracted from the patient file. Prior to data collection, informed consent was taken from patients to allow data extraction from their file. Data collection period was between 01 January and 31 December 2020.

### Data entry and analysis

Data were double entered between January and February 2021, validated and analysed using EpiData (version 3.1 for entry and version 2.2.2.183 for analysis, EpiData Association, Odense, Denmark). Additional analyses were performed using STATA (version 13.0, StataCorp LP USA).

The level of difficulty of laparoscopic cholecystectomy was generated as ‘easy’ or ‘difficult’. Laparoscopic cholecystectomy was defined as ‘difficult’ if the operative time taken was >60 min, if there were presence of biliary leakage, injury to bile duct or artery, or conversion to open cholecystectomy [7]. Post‐operative complications were assessed for the duration until discharge from the hospital.

Unadjusted prevalence ratios (PR) assessed the association of difficult laparoscopic cholecystectomy with selected ultrasonography features of the hepatobiliary system and intraoperative parameters. Risk factors with *p*‐value <0.1 were included in the adjusted analysis (adjusted PR) using log binomial regression model. Adjusted *p*‐value <0.05 were considered significant.

### Ethics approval

This study was performed in accordance with the Declaration of Helsinki, and ethics approval was obtained from the Research Ethics Board of Health, Ministry of Health, Bhutan (REBH/Approval/2019/059 dated 18/12/2019). Informed written informed consent was taken from the study participants as per the consent process approved by the ethics committee. Only anonymized data are presented in this paper.

## RESULTS

There were 134 valid pro forma collected (response rate 95%). There were 97 females (72%). The mean age of the sample was 43 (±SD 13) years. The details of the basic characteristics of the participants are shown in Table 1.

**TABLE 1:** Socio‐demographic characteristics of patients who underwent laparoscopic cholecystectomy at the Jigme Dorji Wangchuck National Referral Hospital, Thimphu, Bhutan, 2020.

Variables	Total		Difficult laparoscopic cholecystectomy	
	*n*	(%)	*n*	(%)
Age group (years)				
18–24	7	(5)	3	(43)
25–34	37	(28)	21	(57)
35–44	38	(28)	26	(68)
45–54	25	(19)	18	(72)
55–64	15	(11)	9	(60)
≥65	12	(9)	6	(50)
Sex				
Male	37	(28)	27	(73)
Female	97	(72)	56	(58)
Body mass index (kg/m^2^)				
Underweight (<18.5)	1	(1)	0	(0)
Normal (18.5–24.9)	48	(36)	25	(52)
Overweight (25–29.9)	62	(46)	40	(65)
Obese (≥30)	23	(17)	18	(78)
Hypertension	18	(13)	14	(78)
Diabetes mellitus	11	(8)	8	(73)
Congestive heart failure	1	(1)	1	(100)
Chronic obstructive pulmonary disease	1	(1)	1	(100)
ASA physical status				
Class I	94	(70)	55	(59)
Class II	39	(29)	27	(69)
Class III	1	(1)	1	(100)
Class IV	0	(0)	0	(0)
Class V	0	(0)	0	(0)
Past history of admission for acute cholecystitis	29	(22)	21	(72)
Current acute cholecystitis	12	(9)	11	(92)
Past upper abdominal surgery	2	(1)	2	(100)
Palpable gall bladder	0	(0)	0	(0)

Abbreviation: ASA, American Society of Anaesthesiologists physical status classification.

The most common indication of laparoscopic cholecystectomy was symptomatic gall stone disease in 129 individuals (96%). The details of clinical and operative details of the participants are shown in Table 2.

**TABLE 2:** Preoperative clinical and operative characteristics of patients who underwent laparoscopic cholecystectomy at the Jigme Dorji Wangchuck National Referral Hospital, Thimphu, Bhutan, 2020.

Variables	Total		Difficult laparoscopic cholecystectomy	
	*n*	(%)	*n*	(%)
**Ultrasonography features**				
Number of stones				
Single	45	(34)	29	(64)
Multiple	89	(66)	54	(61)
Presence of impacted stones	12	(9)	9	(75)
Presence of pericholecystic collection	13	(10)	12	(92)
Presence of contracted gall bladder	11	(8)	5	(45)
Common bile duct diameter				
Normal <7 mm	126	(94)	80	(63)
Dilated ≥7 mm	8	(6)	3	(38)
**Operative details**				
Type of cholecystectomy				
Partial	4	(3)	3	(75)
Total	130	(97)	80	(62)
Duration of surgery				
≤60 min	62	(46)	11	(18)
>60 min	72	(54)	72	(100)
Gall bladder				
Floppy non‐adherent	94	(70)	50	(53)
Empyema	20	(15)	15	(75)
Gangrene	3	(2)	3	(100)
Mass of the gall bladder	1	(1)	1	(100)
Contracted gall bladder	7	(5)	5	(71)
Others	9	(7)	9	(100)
Cystic pedicle				
Thin and clear	50	(37)	22	(44)
Fat laden	57	(43)	36	(63)
Abnormal anatomy or obscured	20	(15)	18	(90)
Impossible to clarify	7	(5)	7	(100)
Adhesions				
None	53	(40)	23	(43)
Simple up to the neck/Hartmann's pouch	22	(16)	12	(55)
Simple up to the body	29	(22)	21	(72)
Dense up to fundus	18	(13)	16	(89)
Dense involving hepatic flexure/duodenum	3	(2)	3	(100)
Dense wrapping the gall bladder or difficult to separate	9	(7)	8	(89)
Spillage of stone	35	(26)	35	(100)
Injury to biliary ducts	0	(0)	0	(0)
Injury to arteries	0	(0)	0	(0)
Conversion to open cholecystectomy	1	(1)	1	(100)

**FIGURE 1:**
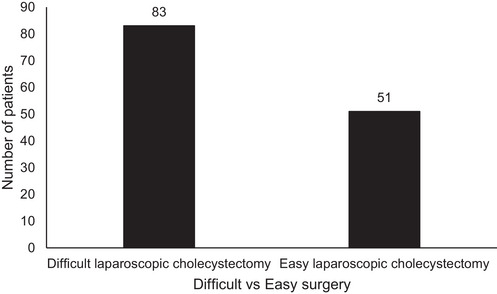
The proportion of difficult versus easy laparoscopic cholecystectomy among patients who underwent surgery at the Jigme Dorji Wangchuck National Referral Hospital, Thimphu, Bhutan, 2020.

The median duration of laparoscopic cholecystectomy surgery was 65 min (IQR 52, 100). As shown in Table 2, the surgery was completed in ≤60 min in 62 individuals (47%), whereas it took more than 60 min in 72 individuals (53%). Based on the definition adopted for this study, ‘difficult laparoscopic cholecystectomy’ was reported in 83 patients (62%) and ‘easy laparoscopic cholecystectomy’ in 51 patients (38%) as shown in Figure 1.

The majority did not have any post‐operative complications (130 cases, 97%). One case (1%) that required intensive care unit admission had acute cholecystitis with septic shock where it was untenable to proceed laparoscopically due to dense adhesions and was converted to open cholecystectomy, subsequently developed abdominal abscess and had a hospital stay of 32 days. The details of the surgery related outcomes are shown in Table 3.

**TABLE 3:** The outcomes of laparoscopic cholecystectomy among patients who underwent laparoscopic cholecystectomy at the Jigme Dorji Wangchuck National Referral Hospital, Thimphu, Bhutan, 2020.

Variables	Total		Difficult laparoscopic cholecystectomy	
	*n*	(%)	*n*	(%)
Post‐operative complications				
None	130	(97)	81	(62)
Bleeding	3	(2)	1	(33)
Bile leak	0	(0)	0	(0)
Collection in Morrison's pouch	0	(0)	0	(0)
Intra‐abdominal abscess	1	(1)	1	(100)
Duration of hospital stay (day(s))				
0	4	(3)	2	(50)
1	28	(21)	15	(54)
2	81	(60)	47	(58)
3	11	(8)	10	(91)
4	4	(3)	3	(75)
5	3	(2)	3	(100)
12–32	3	(2)	1	(33)
Intensive care unit admission	1	(1)	1	(100)
Outcome of surgery				
Discharged alive	134	(100)	83	(62)
Died	0	(0)	0	(0)

In the adjusted analysis, patients having simple adhesions up to the body of the gall bladder, were 1.6 times more likely to encounter difficult laparoscopic cholecystectomy (adjusted PR 1.60, 95% CI 1.04–2.48, *p* = 0.034). The details of the association of factor with difficult laparoscopic cholecystectomy are shown in Table 4.

**TABLE 4:** Ultrasonography and intraoperative factors associated with difficult laparoscopic cholecystectomy among patients who underwent laparoscopic cholecystectomy at the Jigme Dorji Wangchuck National Referral Hospital, Thimphu, Bhutan, 2020.

Factors		Difficult laparoscopic cholecystectomy		Unadjusted analyses		Adjusted analyses	
	Total	*n*	(%)	PR (95% CI)	*p*‐Value	aPR (95% CI)	*p*‐Value
**Ultrasonography features**							
Number of stones							
Single	45	29	(64.44)	Ref			
Multiple	89	54	(60.67)	0.94 (0.72–1.24)	0.666		
Presence of impacted stones	12	9	(75.00)	1.24 (0.87–1.77)	0.243		
Presence of pericholecystic collection	13	12	(92.31)	**1.57 (1.27–1.95)**	**<0.001**	1.08 (0.69‐1.70)	0.725
Presence of contracted gall bladder	11	5	(45.45)	0.72 (0.37–1.39)	0.324		
Dilated common bile duct (transverse diameter ≥7 mm)	8	3	(37.50)	0.59 (0.24–1.46)	0.254		
**Operative details**							
Type of cholecystectomy							
Partial	4	3	(75.00)	Ref			
Total	130	80	(61.54)	0.82 (0.46–1.47)	0.505		
Cystic pedicle							
Thin and clear	50	22	(44.00)	Ref		Ref	
Fat laden	57	36	(63.16)	1.44 (0.99–2.08)	0.056	1.08 (0.72–1.63)	0.697
Abnormal anatomy or obscured	20	18	(90.00)	**2.05 (1.45–2.89)**	**<0.001**	1.36 (0.76–2.45)	0.697
Impossible to clarify	7	7	(100.00)				
Adhesions							
None	53	23	(43.40)	Ref		Ref	
Simple up to the neck/Hartmann's pouch	22	12	(54.55)	1.26 (0.77–2.05)	0.360	1.10 (0.68–1.79)	0.713
Simple up to the body	29	21	(72.41)	**1.69 (1.14–2.44)**	**0.008**	**1.60 (1.04–2.48)**	**0.034**
Dense up to fundus	18	16	(88.89)	**2.05 (1.45–2.90)**	**<0.001**	**1.42 (0.83–2.45)**	**0.202**
Dense involving hepatic flexure/duodenum	3	3	(100.00)				
Dense wrapping the gall bladder or difficult to separate	9	8	(88.89)				

Abbreviations: aPR, adjusted prevalence ratio; CI, confidence interval; PR, prevalence ratio.

## DISCUSSION

The proportion of difficult laparoscopic cholecystectomy was 62% (83 out of 134 cases) and was associated with presence of adhesions up to the body of the gall bladder. This proportion is higher than 21.9% (50 out of 228 cases), 27.6% (83 out of 300 cases) and 32.9% (69 out of 210 cases) reported in India and 26.0% (26 out of 100 cases) reported in Nepal [4, 9, 10]. The operational definitions used in these four articles are comparable to the one adopted in our study. Therefore, based on the standards reported from centres in countries in the region, India and Nepal, the proportion of difficult laparoscopic was 2.1 times higher at the JDW National Referral Hospital, Bhutan.

While we have adopted the time duration of 60 min for completion or conversion to open cholecystectomy, other studies have adopted 90 and 180 min as the cut‐off to define a difficult laparoscopic cholecystectomy [11, 12]. There is a lack of consensus on the definition of difficult laparoscopic cholecystectomy across centres with varying capabilities and volumes of work. In this study, all comparisons were made with centres in the region where training programmes and surgical capabilities may be comparable.

From the 83 patients who had difficult laparoscopic cholecystectomy, 72 individuals had surgeries lasting more than 60 min. Therefore, compared to the regional centres that have reported difficulty in laparoscopic cholecystectomy, the time‐factor is an important area that requires review in our setting. Laparoscopic surgery has a steep learning curve especially among surgeons practicing in resource‐limited trainings where they lack opportunities for practice in skills lab and mentoring from experienced surgeons. As surgeons perform a more number of similar surgeries, there is a significant decrease in the operating time duration, rates of complications such as retained stone, injury to arteries or conversion to open surgery [13].

Although there was longer operating time duration, the rates of complications were comparable to others. There were no incidences of injury to arteries or biliary ducts. The rate of conversion from laparoscopic to open cholecystectomy was very low compared to that of 5.71% (12 out of 210 cases) and 4.9% (24 out of 492 cases) reported elsewhere [10, 14]. At our centre, if the operating surgeon faced difficulties with the procedure, on‐table consults were done with senior surgeons where senior surgeons scrubbed in to complete the surgery and/or considered bail out options such as partial cholecystectomy.

In the adjusted analysis, those who had simple adhesions up to the gall bladder fundus were 1.6 times more likely to have difficult laparoscopic cholecystectomy. Studies lack consensus on the factors that determine difficult laparoscopic cholecystectomy [4, 9, 10]. Therefore, more studies with larger sample size are required to identify such factors that determine operative outcomes.

In our study, there were no mortality incidents related to laparoscopic cholecystectomy, and no cases of bile leak or collections in the Morrison's pouch. There was only one case of intra‐abdominal abscess, and only one patient required intensive care admission. The zero‐mortality rate following laparoscopic cholecystectomy at the JDW National Referral Hospital is an important indicator of the quality of surgical care in the country. Our mortality rate is comparable to a meta‐analysis on the pooled perioperative mortality of surgical conditions in low‐ and middle‐income countries from 2009 to 2014 where cholecystectomy was associated with 0.1% mortality [15].

Providing formal training on laparoscopic surgery is an important area of intervention to standardize surgical practices and ensure patient safety. Providing advanced laparoscopic fellowship training in laparoscopic cholecystectomy is shown to decrease conversion rates for acute biliary pathology [16]. The rate of conversion of laparoscopic cholecystectomy was 8.5% when operated by non‐laparoscopic fellowship‐trained surgeons compared to 1.7% when operated by a trained surgeon [16]. In addition, the trained surgeons were able to better manage the intra‐operative complications and had an overall shorter duration of hospital stay. After the introduction of postgraduate surgical education being provided at the JDW National Referral Hospital since 2014, simulation labs are now available for training on laparoscopic surgeries [17]. The Khesar Gyalpo University of Medical Sciences of Bhutan is collaborating with its international partners in developing programmes for skill transfer in laparoscopy to surgeons practicing in Bhutan. The findings from this study demonstrate the urgent need for investments in skill upgradation of surgeons in laparoscopic and minimally invasive surgeries.

### Limitations

This is a single‐centre study that assessed the factors related to difficult laparoscopic cholecystectomy in the country. Apart from equipment failure, we have assumed that surgeon factors remained similar. However, with new surgeons joining into the health system and with postgraduate trainees involved in performing laparoscopic cholecystectomy [17], a proper analysis of proportions of difficult laparoscopic cholecystectomies at all the surgical centres in the country and a comparison of learning curve of surgeons may offer an understanding on the high proportion of difficult surgery in Bhutan. Another limitation could have resulted from convenience sampling of surgeries that were performed at a referral and a teaching hospital. However, the sample derives strength from patients being referred to this centre from all other districts in the country.

## CONCLUSION

The proportion of difficult laparoscopic cholecystectomy is very high, mostly contributed by a long duration of surgery. Difficult laparoscopic cholecystectomy had significant association with having adhesions up to the body of the gall bladder. The rates of post‐operative complications were minimal with no mortality or injury to bile duct or arteries. This study reflects the present standards of laparoscopic cholecystectomy and the need for appropriate laparoscopy training for surgeons in the country.

## AUTHOR CONTRIBUTIONS

Sangay Wangmo, Sonam Dargay and Sonam ChhodenR were involved in the conception and design of this study. Sangay Wangmo collected, analysed and interpreted the data and drafted the manuscript. Sangay Wangmo, Sonam Dargay and Sonam ChhodenR were involved in critically reviewing the paper and approved the final manuscript.

## CONFLICT OF INTEREST STATEMENT

The authors have no conflicts of interest.

## ETHICS STATEMENT

This study was performed in accordance with the Declaration of Helsinki, and the ethics approval was obtained from the Research Ethics Board of Health, Ministry of Health, Bhutan. Informed written informed consent was taken from the study participants as per the consent process approved by the ethics committee. Only anonymized data are presented in this paper.

## Data Availability

The datasets generated and/or analysed during the current study are available from the corresponding author upon request.
